# Overall Survival Rate of Vietnamese Patients with Colorectal Cancer: A Hospital-Based Cohort Study in the Central Region of Vietnam

**DOI:** 10.31557/APJCP.2021.22.11.3569

**Published:** 2021-11

**Authors:** Duong Dinh Le, Thang Van Vo, Pongdech Sarakarn

**Affiliations:** 1 *Faculty of Public Health, Khon Kaen Univerisity, Khon Kaen province, Thailand. *; 2 *Faculty of Public Health, University of Medicine and Pharmacy, Hue University, Hue city, Thua Thien Hue province, Vietnam. *; 3 *Institute for Community Health Research, University of Medicine and Pharmacy, Hue University, Hue city, Thua Thien Hue province, Vietnam. *; 4 *ASEAN Cancer Epidemiology and Prevention Research Group (ACEP). *

**Keywords:** Colorectal cancer, overall survival, ambidirectional cohort, Vietnamese

## Abstract

**Background:**

This study investigated the overall survival (OS) at 1-year, 3-years, and 5-years after colorectal cancer (CRC) diagnosis and examined the prognostic factors of mortality among patients with CRC in Vietnam’s central region.

**Methods:**

This ambidirectional cohort study included patients newly diagnosed with CRC at a tertiary hospital in Vietnam’s central region between 2013 and 2019. Survival duration was calculated from the surgery date or the first day of CRC-specific treatment until the date of death or the study’s end date, July 31, 2020. Kaplan-Meier methods and log-rank test were used to estimate and compare the OS between the subgroups, respectively. The Cox proportional-hazards (PH) regression analysis was applied to estimate the magnitude of the effects between prognostic factors and outcome.

**Results:**

The median follow-up was 24 months (interquartile range: 13–43 months). The OS rate dropped significantly to 84.7%, 56.19%, and 45.01% at 1-year, 3-years, and 5-years after diagnosis, respectively. The median OS was 48.59 months (39.34 –57.93 months) for the rectum and colon cases. In the multivariate analysis, a higher mortality risk was observed in patients with an advanced-stage CRC (HRadj, 3.04; 95% confidence interval [CI], 1.79–5.18), who were underweight (<18.5 kg/m2; HRadj, 1.65; 95%CI, 1.03–2.65), and had elevated preoperative carcinoembryonic antigen (CEA) level (>5.0 ng/mL; HRadj, 1.63; 95%CI, 1.03–2.59). Additionally, younger patients (<50 years) had a poorer OS than the middle-aged group (60–69 years).

**Conclusion:**

Our findings indicate that <50% of Vietnamese patients with CRC survive until 5-years after diagnosis. Several individual factors that contribute to the poor OS of patients with CRC, including young age, underweight, and elevated preoperative CEA level, should be evaluated and managed. Early diagnoses through active routine examination of or screening programs for high-risk groups should be prioritized.

## Introduction

The burden of colorectal cancer (CRC) has become an important public health issue, which is predicted to increase by 60%, with 2.2 million incident cases and 1.1 deaths by 2030 (Arnold et al., 2017). According to the 2020 GLOBOCAN report, CRC is the second leading cause of cancer-related deaths based on cancer sites worldwide, which is estimated at 9.2% (Ferlay et al., 2020). Although both CRC incidence and mortality are declining in many developed countries, the trend has increased in several Asian countries (Arnold et al., 2017; Wild et al., 2020). Vietnam is a lower- to middle-income country (United, 2020) and is experiencing aging population trends and socioeconomic changes, which are considered the most notable CRC transition markers (Arnold et al., 2017; Hoang et al., 2019). The CRC burden is predicted to double in both incidence and mortality cases in the next two decades in Vietnam (Ferlay et al., 2020).

The survival rate of cancer patients is an essential indicator that reflects the effectiveness of cancer-specific treatment as well as preventive and cancer control programs (Li et al., 2019). In Asian countries, the overall survival (OS) rate of patients with CRC has not improved significantly in the past decades, with the 5-year OS unchanged at approximately 60% (Moghimi-Dehkordi and Safaee, 2012). The 5-year survival rate ranges from 36.87% in Thailand (Phimha et al., 2019) to 73.0% in Japan (Tamakoshi et al., 2017). Nevertheless, the national cancer control program has had limited effects in responding to the demands, particularly for screening programs and cancer-specific treatments (Tran et al., 2016; Hoang et al., 2019; Pham et al., 2019). Many factors contribute to the limitation of cancer epidemiology and survival data, including the lack of surveillance data and the poor quality of cancer survival statistics (Rao et al., 2010; Shin et al., 2012; Hong et al., 2018). To date, the survival rate of CRC has been almost underestimated in Vietnam, and our work is expected to contribute novel knowledge of CRC survival scientific evidence. This study investigated the OS at 1-year, 3-years, and 5-years after diagnosis of CRC and examined the prognostic factors of mortality among Vietnamese patients with CRC in the central region of Vietnam.

## Materials and Methods

*Study design*

This was an ambidirectional cohort study. The initial phase was a retrospective study using a hospital-based cohort from 2013 to 2019. The next phase was a prospective study of the cohort, wherein the identified CRC cases were followed up until the date of death or the study’s end date, July 31, 2020. Survival duration was calculated from the surgery date or the first day of CRC-specific treatment until the date of death or the study’s end date. The vital event status of CRC cases was confirmed from the local community health center where the patients were mostly managed through the national noncommunicable disease program.

*Setting*

This research was implemented in Hue Central Hospital (HCH), a tertiary hospital, established as the highest-level referral hospital for 14 provinces in the central region of Vietnam. The oncology center of HCH is responsible for providing cancer-specific treatment therapies, training, and medical technology implementation of other medical facilities in this area (Hue Central Hospital, 2020). HCH is located in Thua Thien Hue province in the North Central Coast region with a reported population of 1.13 million people in 2019 (General Statistics Office of Vietnam, 2020).

*Participants*

**Figure 1:**
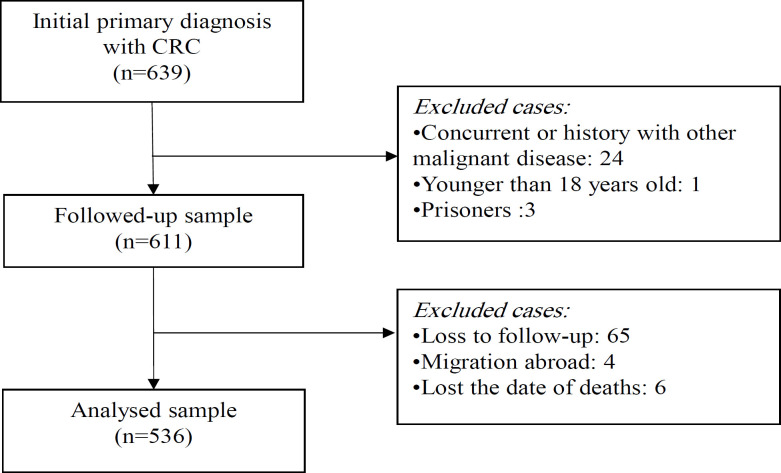
The Eligible Participants

In the beginning, participants with newly diagnosed CRC were selected based on a hospital cohort from January 1, 2013 to December 31, 2019. A total of 639 residential cases were initially diagnosed at baseline and evaluated using the patients’ medical records. In the initial screening, 28 cases were excluded because of other concurrent malignant diseases at enrollment, they were prisoners, or were aged <18 years. Then, 611 confirmed cases were followed up until the date of death or the study’s end date. In our study, 12.2% of cases were withdrawn during the survival analysis, including loss to follow-up (65), emigration (4), and missing data on the exact date of death (6). The eligible participants are shown in Figure 1.

*Measurements*

The patients’ medical record was the primary source of data to collect their baseline characteristics, such as age and sex; clinical characteristics, such as tumor sites; height and weight; preoperative serum carcinoembryonic antigen (CEA) level; and comorbidities, including hypertension, diabetes, and cardiovascular diseases. In this study, CRC staging was classified using the American Joint Committee on Cancer TNM system, seventh edition (2010) (Stephen et al., 2010), which is based on the characteristics of the tumor (T), nodes (N), and metastasis (M). Body mass index (BMI) was calculated based on the patients’ height and weight at the first hospital admission and using the Asian-Pacific classification criteria for BMI grouping (World Health Organization–Regional Office for the Western Pacific, 2000).

The patients’ status was confirmed by the healthcare staff of the local community health center at their residence. In the survival analysis, the outcome was classified into event data as a death case and censored data as alive at the end of the study.

*Statistical analysis*

CRC patients’ demographic and clinical characteristics are presented as mean, standard deviation or median, and interquartile range (IQR) for continuous variables and number and percentage for categorical variables. The Kaplan-Meier estimate was used to depict the OS at 1-, 3-, and 5-year intervals after CRC diagnosis. The median OS with 95% confidence interval (CI) was used to present the survival duration, and the log-rank test was used to compare the OS between subgroups. The Cox proportional-hazards (PH) regression analysis was applied to estimate the magnitude of the effects between prognostic factors and outcome. Only the cases with complete information were selected for the multivariate analysis. The PH assumption was tested using the Schoenfeld residual test. P-values < 0.05 were considered statistically significant.

## Results

*Baseline characteristics of patients with colorectal cancer*

Table 1 presents the participants’ baseline characteristics. The median age was 64 years (IQR: 52–76 years). There was an almost equal percentage between subgroups with respect to several factors: tumor sites, residence, and preoperative CEA level. The median preoperative CEA level was 5.15 ng/mL (IQR: 2.77–13.10). Comorbidities were concurrently observed in 35.63% of patients. Half of the patients had a normal weight, and nearly one-fourth of patients were underweight at enrollment. The median BMI was 20.46 kg/m^2^, and the IQR was 18.37 to 22.43 kg/m^2^. Surgical resection was the most CRC-specific treatment modality, accounting for 85.07%, whereas chemotherapy and radiotherapy were slightly over 50% and 20%, respectively. Among the available TNM stage classifications, advanced stage (III + IV) was observed in 34.33% of cases.

*Overall survival rate and median survival duration*

Table 2 shows the OS according to the time of diagnosis and tumor location. The median follow-up time was 24 months, with an IQR of 13 to 43 months. The OS rate dropped significantly from 84.7% at 1 year to 45.01% at 5 years after diagnosis, and no significant difference was observed between the colon and rectal cancer cases. The median survival duration was 48.59 months for overall CRC, ranging from 39.34 to 57.93 months for colon and rectal cancers, respectively.

*Prognostics factors of overall survival in patients with colorectal cancer*

Table 3 shows the bivariate analysis of prognostic factors and the mortality rate of CRC cases. The OS was significantly different between age groups, disease stages, BMI groups, preoperative CEA levels, and treatment modalities. Nevertheless, sex, comorbidities, tumor sites, and residence had no statistically significant differences in OS.

Table 4 provides the multivariate analysis using the Cox PH regression analysis in a sample of 232 cases. Overall, age group, disease stage, BMI group, and preoperative CEA level were significantly associated with the OS of patients with CRC after adjusting for sex, comorbidities, and treatment modalities. Patients with advanced stage cancers had 3.04 times (HRadj: 3.04; 95%CI: 1.79–5.18) higher risk of mortality rate than those with early stage cancers. Middle-age groups (aged 60–69 years) were found to have a lower risk of death (HRadj, 0.47; 95%CI, 0.24–0.91) than the youngest age group (aged <50 years). In addition, CRC patients who were underweight (BMI <18.5 kg/m^2^) and had an elevated preoperative CEA level had a higher mortality risk at 1.65 and 1.63 times, respectively.

**Table 1:** Baseline Characteristics of Colorectal Cancer Patients

Characteristics	Number	Percentage (%)
Age groups (years)		
< 50	112	20.9
50 - 59	103	19.22
60 - 69	124	23.13
70 - 79	109	20.34
≥80	88	16.42
Gender		
Male	295	55.04
Female	241	44.96
Residence		
Urban	239	44.59
Rural/ mountainous	297	55.41
Comorbidities		
Hypertension	153	28.54
Diabetes	60	11.19
Cardiovascular diseases	41	7.65
BMI groups		
Underweight (<18.5 kg/m^2^ )	131	24.44
Normal (18.5-<23.0 kg/m^2^)	266	49.63
Overweight (23.0-<25.0 kg/m^2^)	52	9.70
Obesity (≥25 kg/m^2^)	41	7.65
Unknown	46	8.58
Tumor sites		
Colon	278	51.87
Rectum	258	48.13
Disease stages		
Stage I	32	5.97
Stage II	111	20.71
Stage III	114	21.27
Stage IV	70	13.06
Unknown stage	209	38.99
Preoperative CEA level		
Non elevated (≤5ng/ml)	197	36.75
Elevated (>5 ng/ml)	207	38.62
Unknown	132	24.63
Treatment modalities		
SR only	191	35.63
SR +CT or RT	195	36.38
SR + CT + RT	70	13.06
CT/ RT without SR	36	6.72
Not at all	44	8.21

IQR, interquartile range; BMI, body mass index; CEA, Preoperative serum carcino-embryonic antigen; SR, surgical resection; CT, chemotherapy; RT, radiotherapy.

**Table 2:** Overall Survival Rate and Median Survival duration of CRC Patients

Tumor location	Overall survival rate, % (95%CIs)			Median survival duration (95%CIs)
	1-year	3-year	5-year	
Colorectal cancer	84.7	56.19	45.01	48.59
(n=536)	(81.34-87.50)	(51.24-60.84)	(39.43-50.42)	(37.34-63.47)
Colon cancer	83.67	58.84	49.6	57.93
(n=278)	(78.75-87.54)	(52.15-64.91)	(42.13-56.61)	(42.66 – NA)
Rectal cancer	85.8	52.78	38.91	39.34
(n=258)	(80.85 – 89.55)	(45.24-59.76)	(30.49-47.23)	(33.44-49.44)

CIs, confidence interval; NA, not applicable

**Table 3:** Univariate Analysis of Baseline Characteristics and Survival of Colorectal Cancer Patients

Factors	Total	Death	Events/ 1000 months	Median OS (months)	Crude HR (95%CI)	p-value*
Age groups (years)						<0.001
< 50	112	48	12.68	56.56	ref	
50 - 59	103	33	10.65	68.56	0.81 (0.52-1.26)	
60 - 69	124	39	9.90	68.85	0.78 (0.51-1.19)	
70 - 79	109	61	18.51	34.39	1.43 (0.98-2.09)	
≥ 80	88	49	20.5	25.84	1.57 (1.05-2.34)	
Gender						0.563
Female	241	108	14.53	49.21	ref	
Male	295	122	13.45	47.73	0.93 (0.72-1.20)	
Comorbidities						0.851
No	345	154	14.06	47.74	ref	
Yes	191	79	13.7	55.25	0.97 (0.74-1.28)	
Tumor sites						0.313
Colon	278	117	12.86	57.93	ref	
Rectum	258	113	15.25	37.87	1.14 (0.88-1.48)	
Residence						0.98
Urban	341	144	13.87	49.21	ref	
Rural/ mountainous	195	86	13.97	47.74	1.003 (0.77-1.31)	
TNM Stages						<0.001
Stage I	32	4	3.20	-	ref	
Stage II	111	26	7.50	-	2.25(0.79-6.46)	
Stage III	114	41	11.90	49.21	3.60 (1.29-10.07)	
Stage IV	70	53	39.10	16.95	11.88 (4.29-32.89)	
Unknown stage	209	109	15.10	40.23	4.77 (1.76-12.94)	
BMI group (n=490)						0.002
Normal	266	103	12.86	48.59	ref	
Overweight	52	11	6.61	NA	0.53 (0.29-0.1.03)	
Obesity	41	21	20.28	31.44	1.53 (0.96-2.45)	
Underweight	131	73	18.30	34.79	1.48 (1.10-2.00)	
Preoperative CEA level (n=404)						0.001
Non elevated	197	71	9.60	80.95	ref	
Elevated	207	101	16.71	34.39	1.70 (1.25-2.31)	
Treatment modalities						
SR only	191	72	10.70	83.93	ref	<0.001
SR +CT or RT	195	84	13.96	49.44	1.24 (0.90-1.70)	
SR + CT + RT	70	28	14.33	42.66	1.20 (0.78-1.87)	
CT/ RT without SR	36	18	24.43	31.15	2.07 (1.13-3.48)	
Not at all	44	28	26.13	21.31	2.34(1.51-3.62)	

HR, hazard ratio; ref, reference group; NA, not applicable; SR, surgical resection; CT,chemotherapy; RT, radiotherapy; *Log-rank test.

**Table 4:** Factors Affecting Mortality of CRC Patients in Multivariate Analysis

Factors	Adjusted HR* (95% CI)	p	Test proportional assumption		
			Rho	Chi2	p
Age groups (years)					
<50	ref				
50-59	0.99 (0.55-1.79)	0.969	0.001	0	0.993
60-69	0.47 (0.24-0.91)	0.025	-0.168	2.6	0.107
≥70	1.23 (0.68-2.23)	0.484	-0.037	0.12	0.726
Stage groups					
I + II (early stage)	ref				
III+IV (advanced stage)	3.04 (1.79-5.18)	<0.001	0.011	0.01	0.913
BMI groups					
Normal	ref				
Overweight	1.04 (0.43-2.49)	0.953	-0.104	1	0.317
Obesity	1.48 (0.65-3.32)	0.355	-0.01	0.01	0.924
Underweight	1.65 (1.03-2.65)	0.037	-0.04	0.16	0.693
Preoperative CEA level					
Non elevated	ref				
Elevated	1.63 (1.03 – 2.59)	0.038	-0.094	0.82	0.365

*) Adjusted for tumor sites, treatment modalities, sex, and comorbidities. Harrell's C= 0.7102, global test =0.2839. Multivariate analysis was performed in 232 samples.

## Discussion

Worldwide, the incidence of CRC continues to rise (Rawla et al., 2019), and the trend patterns of the disease correlate with the present human development levels, which may reflect the adoption of more Western lifestyles (Arnold et al., 2017). In 2020, more than one in two new CRC cases occurred in Asian countries (Ferlay et al., 2020). In this study, we focused on the OS rate based on the limited quality of mortality data; in particular, the cause of death in our nation (Hong et al., 2018; Pham et al., 2019).

The results of this study indicated that a reduction in the OS rate was apparent in Vietnamese patients with CRC. The OS declined significantly by approximately 40% from 84.7% at 1 year to 45.01% at 5 years after diagnosis. In our samples, the 5-year OS of colon cancer patients was 49.6% compared to 38.91% of rectal cancer patients, and no significant difference was found between the tumor sites (Table 2). Survival rates were studied previously (Coleman et al., 2011), with the 5-year survival rate at 16% in Ghana (Agyemang-Yeboah et al., 2018), 44% in Spain, 43.8% in Martinique (France) (Joachim et al., 2019), 63.5% in Brazil (Aguiar et al., 2020), and 66.6% in the United States (Siegel et al., 2017). This variation was observed within a region, that is, the 5-year survival rate in Eastern Mediterranean countries was 57.26% (50.43%–64.10%), which varied from 29.5% in Libya to 95% in Lebanon (Nikbakht et al., 2020). We found that the 5-year OS of Vietnamese patients with CRC was approximately 60% lower than those in Asian countries (Moghimi-Dehkordi and Safaee, 2012). Our findings are equivalent to those of neighboring countries, such as Thailand (Phimha et al., 2019; Kittrongsiri et al., 2020), Malaysia (Magaji et al., 2017), and Brunei (Leong et al., 2020). In the literature, the survival rate can reflect the quality of healthcare services, such as an accurate diagnosis, effective management, and treatment of cancer patients (Coleman et al., 2011). In this study, the median follow-up duration was 24 months, from 7 days to 90 months. In general, half of the CRC patients survived after 48 months (median OS, 48.59; 95%CI, 37.34–63.47), which was comparable to the median OS of Malaysian citizens (42.00 months, 95%CI: 35.42–48.58) (Magaji et al., 2017), but lower than that in Brunei (57.0 months, 95% CI: 42.4–79.9) (Leong et al., 2020).

In terms of prognostic factors of the mortality rate of CRC patients, the disease stage is consistently considered an independent risk factor of mortality in the literature (Maringe et al., 2013; Wild CP et al., 2020). In our results, cancer patients in an advanced stage (stage III + IV, 34.33%) had a higher mortality rate than those in the early stage (26.68%) (Table 1). Although a high number of patients were found without a disease stage (39%), this rate is comparable with other studies in neighboring countries (Magaji et al., 2017; Phimha et al., 2019). In this study, the disease stage was observed to be statistically significant in bivariate and multivariate analyses. The 5-year OS of patients with an early diagnosis was 70.6% (95%CI: 58.6–78.8) compared to 31.9% (95%CI: 22.6–41.6) in the advanced stage. In addition, CRC patients with an advanced stage had a 3.04 times increased risk of mortality (HR, 3.04 [1.79–5.18]) (Table 4), which are similar to those of previous studies (Yuan et al., 2013; Magaji et al., 2017; Phimha et al., 2019).

The relationship between age at diagnosis and outcome of CRC patients has been consistently reported in many previous studies (Van et al., 2015; Sharkas et al., 2017; Gabriel et al., 2018; Kittrongsiri et al., 2020; Leong et al., 2020). Currently, the aging population trend is occurring worldwide, including Vietnam. In our sample, approximately 60% of patients were older (≥60 years), and a lower median OS was reported in the older group, particularly those aged ≥70 years. Older patients had an obviously increased mortality risk; however, a slightly higher rate of poor outcome was also observed in younger patients (Table 3). Several studies have shown that young patients with CRC are diagnosed late and show poor pathology (Chou et al., 2011; Berut et al., 2013). In this study, we found that the middle-aged group (aged 60–69 years) had a lower risk of mortality than the younger age group (aged <50 years) (HR, 0.47; 95%CI, 0.24–0.91). These results are similar to those of other studies in China: the 5-year survival differed between age groups and younger patients had a slightly lower OS than those aged 60–74 years (Yuan et al., 2013).

Our findings showed that half of the cases had a normal weight (18.5 to <23.0 kg/m^2^) at baseline. The OS was significantly different between the BMI groups (Table 3), and the median OS was 48.59, 31.44, and 34.79 for the normal, obese, and underweight groups, respectively. In the multivariate analysis, a poor effect of BMI was statistically significantly different on the OS of the underweight group (HR, 1.65; 95%CI, 1.03–2.65). In our cohort, an underweight status was commonly reported at diagnosis (24.44%). To date, the effects of BMI on the survival rate of CRC patients are still inconsistent (Wang et al., 2017). According to a recently published article, the authors found that BMI at diagnosis was associated with all-cause and CRC-specific mortalities in a nonlinear fashion. Underweight patients had a higher mortality risk (HR, 2.65; 95%CI, 1.63–4.31) (Kroenke et al., 2016). Our findings maintained the relationship between the OS and BMI groups following a U-shaped pattern (Wang et al., 2017). This result was similar to the findings of a systematic review; underweight patients had an increased risk of all-cause mortality (RR, 1.43; 95%CI, 1.26–1.62), and overweight patients did not have any significant risk of any outcomes (Doleman et al., 2016).

Preoperative serum CEA, a glycoprotein of bullous pemphigoid antigen ~180 kDa, is frequently expressed in colorectal tumors (Hayat, 2009). In our samples, more than 75% of patients were evaluated for CEA level upon admission, and more than half of them had elevated serum CEA levels (>5 ng/mL) (Table 1). CRC patients with elevated preoperative CEA levels had a higher risk of mortality than those with nonelevated CEA levels (HRadj, 1.63; 95%CI, 1.03–2.59). The role of CEA level in our findings are consistent with the findings of previous studies. Higher CEA levels are a significant prognostic factor for OS and disease-free survival in Vietnam (Nguyen et al., 2019). In a tertiary hospital cohort in Malaysia, the preoperative CEA level was significantly associated with the 5-year survival rate of patients with CRC (Magaji et al., 2017). Similarly, the 5-year survival of Chinese patients differed significantly with serum CEA levels (Yuan et al., 2013).

In conclusion, our findings indicate that the OS of Vietnamese patients with CRC was lower than that in Asian countries and developed nations. Less than 50% of patients survive 5-year after a CRC diagnosis. The disease stage is a critical prognostic marker of survival rate, and late-stage diagnosis remains high in Vietnamese patients. Therefore, we recommend that priority strategies should be encouraged for early diagnosis through active examination of and screening programs for the high-risk group. Other individual factors that contribute to the poor OS of CRC patients, such as the young age, underweight, and elevated preoperative CEA level, should be evaluated and managed.

## Author Contribution Statement

DLD, TVT, and PS contributed to the development of the study design and conceptual framework of this research. TVT and PS co-supervised and revised the manuscript. DLD prepared the draft manuscript, collected data, analyzed, and interpreted the results. All authors have read and approved the final manuscript.

## Funding statement

The study was funded by the Institute for Community Health Research, University of Medicine and Pharmacy, Hue University, Vietnam. 

## Conflict of interest

The authors declare that there is no conflict of interest.

## Ethical consideration 

The study was approved by the Ethical Committee of Hue University of Medicine and Pharmacy, Vietnam (no. H2019/430), and Khon Kaen University Ethics Committee for Human Research, Thailand (no.HE632177).

## Availability of data

This study is part of the approved student PhD thesis in Epidemiology and Biostatistics Program, Khon Kaen University, Thailand and data is available upon request.
